# Serological evidence for human cystic echinococcosis in Slovenia

**DOI:** 10.1186/1471-2334-8-63

**Published:** 2008-05-09

**Authors:** Jernej Logar, Barbara Soba, Tadeja Kotar

**Affiliations:** 1 Department of Parasitology, Institute of Microbiology and Immunology, Medical Faculty, University of Ljubljana, Ljubljana, Slovenia; 2 Department of Infectious Diseases and Febrile Illnesses, University Medical Centre Ljubljana, Ljubljana, Slovenia

## Abstract

**Background:**

Cystic echinococcosis (CE) is caused by the larva of tapeworm *Echinococcus granulosus*. Dogs and other canids are the primary definitive hosts for this parasite. CE may develop after accidental ingestion of tapeworm eggs, excreted with the feces of these animals. In the intestine, the larvae released from the eggs are nested in the liver, lungs or other organs of livestock as intermediate hosts and humans as aberrant hosts. The aim of this study was to examine serologically whether some of the patients in Slovenia, suspected of CE by imaging findings in the liver or lungs had been infected with the larva of *Echinococcus granulosus*.

**Methods:**

Between January 1, 2002 and the end of December 2006, 1323 patients suspected of having echinococcosis were screened serologically by indirect haemagglutination assay (IHA). For confirmation and differentiation of *Echinococcus *spp. infection, the sera of IHA-positive patients were then retested by western blot (WB).

**Results:**

Out of 127 IHA-positive sera, 34 sera were confirmed by WB and considered specific for CE. Of 34 sera of CE-positive patients sera, 32 corresponded to the characteristic imaging findings of a liver cysts and 2 to those of lung cysts. The mean age of CE-positive patients was 58.3 years. No significant differences were found between the CE-positive patients in regard to their sex.

**Conclusion:**

In the study, it was found out that CE was mostly spread in the same area of Slovenia as in the past, but its prevalence decreased from 4.8 per 10^5 ^inhabitants in the period 1956–1968 to 1.7 per 10^5 ^inhabitants in the period 2002–2006. In spite of the decreased prevalence of CE in the last years, it is suggested that clinicians and public health authorities, especially in the eastern parts of Slovenia where the most CE patients come from, should pay greater attention to this disease in the future.

## Background

Human cystic echinococcosis (CE) is caused mainly by the larva of tapeworm *Echinococcus granulosus*. *E. granulosus *occurs worldwide. It is 2–7 mm long and is harboured in the intestine of definitive hosts, typically dogs and other canids which are infected by the ingestion of offal containing larval stage of *E. granulosus*. The tapeworm eggs are excreted with the faeces of these animals. Following accidental ingestion of these eggs, CE in larval stage may develop in ungulates and sometimes in humans as intermediate hosts, usually in the liver or lung [1]. The aim of this study was to examine serologically whether the Slovenian patients suspected of having cystic echinococcosis had been infected by the larvae of *E. granulosus*.

## Methods

Between 1 January 2002 and the end of December 2006, 1323 patients suspected of having CE (because their ultrasound or computerised tomography scans showed round or oval lesions of different size in the liver or lung) were serologically examined for this disease at the Department of Parasitology, Institute of Microbiology and Immunology, Medical Faculty of Ljubljana. The sera were obtained from the patients of both genders and different ages, and from different parts of Slovenia. The sera were screened by an indirect haemagglutination assay (IHA) (Cellognost-Echinococcosis, Dade Behring, Marburg, Germany).

IHA-low positive titres of 1:32 to 1:128 or higher could only be accepted as positive for echinococcosis if confirmed in conjunction with a second serological method, e. g. immunofluorescent test, ELISA, western blot (WB). For confirmation and differentiation between CE and alveolar echinococcosis (AE) caused by *Echinococcus multilocularis*, all IHA sera with low, mid-range, and high titres were retested by WB IgG (LDBIO Diagnostics, Lyon, France). According to the manufacturer's interpretation data and test evaluation by Liance et al., 2000, immunoblot patterns P1 with only a 7 kDa band, and P2 with a band at 7 kDa and at least a diffuse band at 16–18 kDa are specific for CE. Pattern P3 with at least one band at 26–28 kDa plus two sharp bands at 16 and 18 kDa is the most specific pattern for AE. Pattern P4 which includes only one band of 26 to 28 kDa is also c. 88% specific for AE. Pattern P5 with the bands at 7 kDa and 26–28 kDa and without additional intermediate bands cannot be used to discriminate between the two species [2]. Because of possible cross-reactivity with neurocysticercosis, *E. granulosus-*positive sera were retested using an immunoblot assay Cysticercosis WB IgG (LDBIO Diagnostics).

The research has been performed with the approval of the National Medical Ethics Committee of the Republic of Slovenia. Concerning the statements of the NMEC, in true non-interventional, purely observational studies, classical informed consent is not required, similarly to the situation with epidemiological studies, which do not involve any intervention on or interaction with patients and where the right to confidentiality/private life is not interfered with.

## Results

In 127 of 1323 sera from the patients with suspected echinococcosis, antibodies to *Echinococcus *spp. with low to high titres were detected by IHA. Out of IHA-positive sera that were not confirmed by WB, 74 sera were positive in the low titres 1:32–1:128, 4 were positive in the titre 1:256, and one in the titre 1:512. Of 127 IHA-positive sera, only 48 sera were positive for echinococcosis by WB. Eight sera with pattern P4 were already confirmed in our previous study to be positive for alveolar echinococcosis. Six sera with pattern P5 with no possibility to discriminate between two species were not studied further [3]. Of 48 sera, 32 sera of patients with cysts in the liver (3 sera were IHA-positive in the titre 1:64, 4 in the titre 1:128, 11 in the titre 1:256, 6 in the titre 1:512, 2 in the titre 1:1024, 1 in the titre 1:2048, 2 in the titre 1:4096 and 3 sera in the titre 1:8192) and two sera of patients with lung echinococcosis (1 serum was positive in the IHA titre 1:256 and other in the titre 1:512) appeared to be positive for CE caused by *Echinococcus granulosus*, as the antibodies in these sera bound specifically to antigen at 7 kDa only or to 7 kDa and diffuse band at 16–18 kDa. Twenty four sera were from women, while 10 sera were from men. The mean age of detected CE positive patients was 58.3 years. CE-positive serological results of the patients corresponded to the imaging findings (univesicular, round or oval cysts with well defined wall contours and the size ranging between 1 and 10 cm) in their liver and lungs. All CE positive sera tested by the immunoblot assay were negative for cysticercosis.

## Discussion

It is known that the initial phase of primary Echinococcus infection is always asymptomatic. Small cysts not inducing major disease may remain asymptomatic for many years, if not permanently. The incubation period of CE is unclear, but probably lasts for many months to years. The infection may become symptomatic if the cysts either rupture or exert a mass-effect. The cystic larva develops mainly in the liver (70%), also in the lungs (20%) and 10% of cysts can occur almost anywhere in the body [4-6]. In our study, CE was detected more frequently in the liver than in the lungs, but it is impossible to compare these results because more examined sera were obtained from the patients with visceral than with lungs disorders. From the data in the literature, CE may occur in the subjects younger than 1 year to those older than 75 years [1]. The age of our CE-positive patients ranged from 24 to 86 years, with the mean age of 58.3 years. No significant differences were found between the CE-positive patients in regard to their sex.

**Figure 1:**
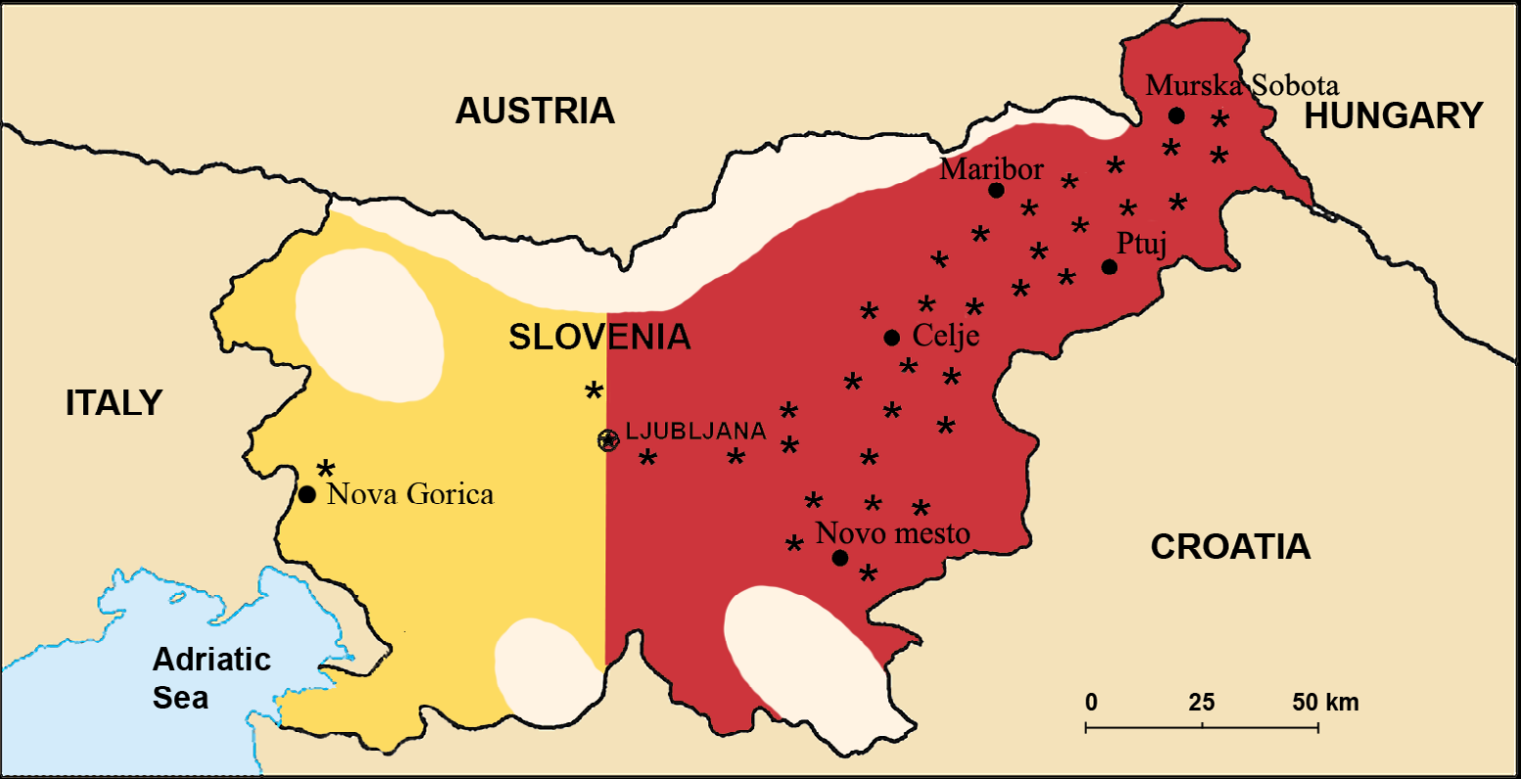
**Regions in Slovenia where the patients' sera tested for CE came from**. From the yellow coloured area, the sera of 483 patients were tested for CE of which 2 (0.4%) sera were confirmed positive (*) for CE, and the sera of 481 (99.6%) patients were CE negative. From the red (eastern) coloured area, the sera of 840 patients were tested for CE of which 32 (4%) were CE-positive (*) and 808 (96%) CE-negative. Places not coloured are not populated due to mountains and/or forests.

*E. granulosus *has a worldwide geographical distribution. It is found on all continents, with the highest prevalence in parts of the Russian Federation and adjacent independent states, China, north and east Africa, Australia, and South America. In Europe, it is present in every country or region (the annual incidence of hospital cases of human CE vary between <1 and >8 per 10^5 ^population) with the exception of Ireland, Iceland and Denmark. It is most intensely endemic in parts of Spain, southern Italy and Sardinia. Reports from several countries also provide documented evidence of an increase in the CE incidence in recent years. For example in Bulgaria the annual incidence of CE in children increased from 0.7 per 10^5 ^in 1971–1982 to 5.4 in 1995. Also in the northern part of Italy, where this disease plays a minor role, the average yearly human CE-incidence during 2003–2005 was between 9.4 and 5.6 per 10^5 ^inhabitants [4,5,7,8]. In 1956 to 1968, the prevalence of CE in the total population of Slovenia, was 4.8 per 10^5 ^inhabitants [9]. In this study, it was found out that CE was mostly spread in the same area, i.e. eastern part of the country, as in the past years, but its prevalence for the five-year period was lower, just about 1.7 per 10^5 ^inhabitants, with the mean annual incidence of 0.34 cases per 10^5 ^inhabitants (Figure 1).

Most forms of human CE are transmitted in domestic lifecycles involving dogs and livestock. The most known is the sheep-dog cycle. In the last years, it has been recognised by genetic characterisation that, in the genus of Echinococcus, there are distinct species and strains (e.g. sheep, pig, cattle, cervid, camel strain) based on morphology, host specificity and molecular characteristics [10,11]. In the 1970's, Brglez estimated that the pig (its breeding is also nowadays the most wide-spread in the eastern parts of the country) was the most important intermediate host in epidemiological and epizootiological conditions of CE in Slovenia [9]. Because it is impossible to allocate the detected CE-positive cases to any of the above mentioned forms by serological methods used in our study, we can only conclude that our patients have been infected by one of species/strains of *Echinococcus granulosus *complex. It is suggested that the decrease of CE reported in this study is probably due to the controlled and reduced slaughtering by the breeders themselves, better general and sanitary attitude of livestock breeders, especially pig breeders, provision of safe drinking water, and control and anthelmintic treatment of dog population.

## Conclusion

The results of this study suggest that CE is still a public health problem in Slovenia, predominantly in the eastern areas of the country; therefore, clinicians and health authorities should pay grater attention to CE and should make additional efforts to decrease or even eliminate this disease in the future.

## Competing interests

The authors declare that they have no competing interests.

## Authors' contributions

JL planned the study, compiled the results and wrote the manuscript. BS carried out the serological assays. TK participated in coordination and management of the patients' clinical data. All authors read and approved the final manuscript.

## Pre-publication history

The pre-publication history for this paper can be accessed here:


